# Investigating perceptions and attitude toward telenursing among undergraduate nursing students for the future of nursing education: a cross-sectional study

**DOI:** 10.1186/s12912-024-01903-2

**Published:** 2024-04-08

**Authors:** Minji Mun, Subin Choi, Kyungmi Woo

**Affiliations:** 1 College of Nursing, Seoul National University, 103 Daehak-Ro, Jongno-Gu, Seoul Republic of Korea; 2 Research Institute of Nursing Science, College of Nursing, Seoul National University, 103 Daehak-Ro, Jongno-Gu, Seoul 03080 South Korea

**Keywords:** Telenursing, Perception, Attitude, Nursing students, Education, Telemedicine

## Abstract

**Background:**

Telenursing is poised to emerge as a novel healthcare delivery system in the digital age. Hence, understanding nursing students' perspectives and readiness is pivotal for its effective implementation. This study investigated nursing students' perceptions regarding, and attitudes toward, telenursing and the factors that influenced their attitudes based on the technology acceptance model.

**Methods:**

This study used a cross-sectional descriptive approach. The participants consisted of 188 nursing students (first to fourth year) enrolled in the College of Nursing in Korea. Differences in attitudes toward telenursing were analyzed using independent t-test and one-way analysis of variance. Pearson’s correlation coefficient was used to examine the correlations between the main variables. Factors that influenced attitudes toward telenursing were analyzed using multiple regression.

**Results:**

Of the participants, 65.4% lacked substantial awareness of telenursing and 19.1% had prior telenursing experience. Although prospects on telenursing indicated that 90.4% had an optimistic view, face-to-face nursing was heavily preferred for both satisfactory and favored healthcare delivery. Many cited the Internet as their source of knowledge, and only 18.6% had received telenursing education. Attitude toward telenursing was significantly more positive among those with experience of telenursing, telenursing observation in clinical practice, and telenursing education exposure. The regression model was statistically significant (F = 67.445, *p* < .000). Factors, such as perceived usefulness, social influence, innovativeness, and self-efficacy, influenced attitudes toward telenursing.

**Conclusions:**

Nursing students exhibited a lack of substantial awareness of telenursing; however, they simultaneously displayed a positive outlook. This lack of comprehensive understanding could stem from the absence of formal education in telenursing. Understanding and utilizing the potential of telenursing could be significantly aided by nursing students' education and knowledge. Thus, it is necessary to include telenursing education in the nursing curriculum. The skills and knowledge required for telenursing clinical practice can be developed through telenursing education. Such preparedness will affect nurses’ attitudes and intentions and the quality of telenursing offered to patients in the future.

**Supplementary Information:**

The online version contains supplementary material available at 10.1186/s12912-024-01903-2.

## Background

With the advancement of information and communication technology (ICT) and onset of the COVID-19 pandemic, “non-face-to-face” interactions have become prevalent worldwide [1]. This shift has led to a rapid surge in the use of telehealth services. Hence, telenursing has gained significant attention as an innovative paradigm in nursing [2]. Telenursing is defined as “the use of telecommunication and information technology for nursing practice from a distance” [3]. It encompasses a spectrum of activities, ranging from nurses counseling patients over the phone to delivering further complex nursing services at the patient's home via cameras and remote monitoring devices [4]. This approach transcends spatial limitations and enables nurses to promptly offer services based on the patient's needs [5]. Telenursing is positioned to emerge as a pioneering healthcare delivery system in the digital age and poised for substantial growth [6].

During the COVID-19 pandemic, South Korea temporarily allowed teleconsultations, triggering significant discussions on legalizing telemedicine [7]. Subsequently, a national pilot project permitting non-face-to-face consultations during holidays and nighttime has been underway since December 2023, indicating substantial changes in South Korea's telemedicine policies for the future. With robust ICT infrastructure [8] and ownership of the necessary technological framework [9], South Korea has the potential for advancing telehealth [10]. Given telehealth's global trend, it is foreseeable that South Korea will soon embrace the introduction and utilization of telenursing. There are currently no regulations regarding telemedicine for nurses, and no discussions on telenursing have been conducted at all. However, previous studies have shown that 43.1% of home healthcare nurses have experienced telenursing [11], and the proportion of military nurses who have experienced telemedicine is 30.1% [12], indicating that telenursing frequently occurs undetected. Once the legal and institutional barriers are addressed, integration and activation of telenursing, can be expected [13]. In preparation, enhancing future nursing professionals' understanding of telenursing and establishing a robust human infrastructure is pivotal. Receptiveness and adaptability within the nursing domain emerge as crucial factors in shaping the quality of healthcare services through telenursing [14]. Studies have indicated that the lackluster performance of pilot telemedicine collaboration projects in Korea resulted from overlooking the factors that influenced medical staff's acceptance of new technologies, such as perceived ease of use, perceived usefulness, and attitude [15]. Therefore, assessing nursing professionals’ attitudes and perceptions is imperative for the stable integration of innovative nursing technologies.

Nursing students will play crucial roles as essential practical personnel [16]. Understanding undergraduate nursing students' perspectives and receptiveness toward telenursing can significantly influence its integration in the upcoming shift to a telehealth era. However, limited research has delved into the status of nursing students concerning telenursing. Existing research on nursing and medical students in countries such as the United States and Poland has suggested that their perspectives and attitudes toward telehealth and telenursing are positive. However, the studies also revealed a lack of adequate education and perception of knowledge gaps [17–21]. To the best of our knowledge, limited studies have examined the perceptions and attitudes of Korean nursing students toward telenursing, particularly following the COVID-19 pandemic.

Therefore, this study aimed to assess Korean nursing students' perception and attitude toward telenursing, explore their intention to utilize telenursing services in their future practice, and evaluate their perspective on the necessity of integrating telenursing services into the national healthcare system. Additionally, we investigated nursing students' educational status regarding telenursing, and aimed to offer insights into the prospect of telenursing education by examining students' educational needs.

### Research framework

This study is based on the Technology Acceptance Model (TAM), proposed by Davis (1989) [22]. The TAM explores the causal relationships among beliefs, attitudes, and behaviors in the process of technology adoption. It also explores how a user's beliefs affect their attitude, which, in turn, influences their intention to use the technology. This intention ultimately drives actual usage. Specifically, perceived usefulness, belief in technology enhancing job performance, perceived ease of use, and belief in minimal effort required shape a user's attitude, impact their intention to use technology, and subsequently, their actual usage [22]. While various theories attempt to explain and predict technology acceptance, the TAM is recognized as a specific robust model in explaining and predicting technology usage [23]. Previous studies [24, 25] have demonstrated the utility of the TAM across diverse nursing-related phenomena. The inclusion of the “attitude” variable distinguish TAM from other models. Thus it is useful for elucidating the introduction and utilization of new technologies in the nursing field. Building on TAM, our study emphasizes “attitude” variable as pivotal in predicting technology adoption. Attitude encompasses beliefs and significantly impacts one’s cognition and behavior.

A previous meta-analysis study found that the TAM models that incorporated the attitude variable demonstrated higher explanatory power compared with those without [26]. Thus, by expanding on the TAM and incorporating external variables, we established a research framework to identify the factors that influenced nursing students' attitudes toward telenursing.

**Fig. 1:**
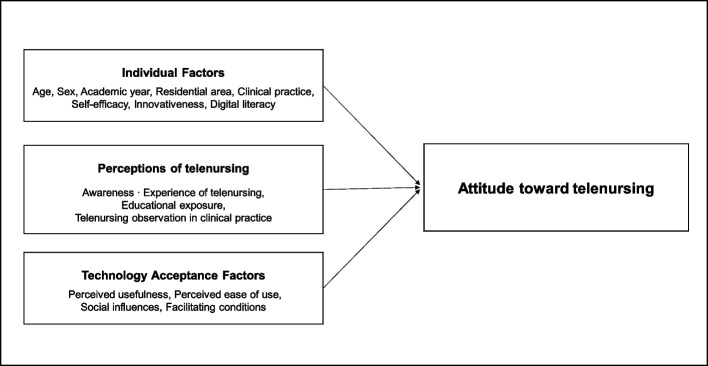
The research framework of this study

Our study developed a research framework, which expanded on the TAM, to identify factors that affected nursing students' attitudes toward telenursing. We considered individual factors, technology acceptance factors, and perceptions of telenursing as independent variables, and attitude toward telenursing as the dependent variable (Fig. 1). Specifically, individual factors comprised demographic characteristics, such as age, sex, academic year, residential area, and clinical practice. Furthermore, self-efficacy, innovativeness, and digital literacy were also incorporated, as previous research identified their influence on technology acceptance [27]. Technology acceptance factors consisted of perceived ease of use and usefulness from the TAM theory, complemented by social influence and facilitating conditions from the Unified Theory of Acceptance and Use of Technology [28]. Perceptions of telenursing were defined as awareness of and experience with telenursing, educational exposure, and observation of telenursing during clinical practice.

## Methods

### Study design

A cross-sectional descriptive survey was conducted using an online questionnaire to investigate attitudes toward telenursing and analyze the factors that influenced them. The participants were nursing students (first to fourth year) enrolled in the College of Nursing in Korea. To determine the required sample size, G-Power 3.1.9.4 software was used. It was calculated with a significance level of 0.05, power(1-β) of 0.8, a medium effect size of 0.15 [29], and 16 variables for the multiple regression analysis, which yielded 143 participants. We considered the potential dropout rates and incomplete responses and added an additional 20%, which resulted in a sample size of 174 [30]. Before the main survey, a pilot test was conducted with 10 participants to verify the questionnaire's validity. The pilot test results were excluded from the final analysis. The main survey was completed by 188 participants.

### Measures and scales

To identify the factors that influenced nursing students' attitudes toward telenursing, a structured questionnaire that consisted of 51 items was employed. Survey items were formulated by referencing prior research on perceptions of telenursing along with studies that investigated acceptance factors based on the TAM theory. All measurements were approved for use by their original authors [27, 31–35]. Additionally, all survey items were adapted to the undergraduate nursing student in Korea and reviewed by a nursing professor in community nursing.

#### Individual factors

Based on previous research on nursing students [21], we developed five questions to explore demographic characteristics, such as sex, age, academic year, residential area, and clinical practice. Self-efficacy was measured via a tool adapted from prior research [31, 32] and comprised two items (e.g., “I could understand and utilize telenursing system.”). Cronbach’s alpha was 0.906. In this study, innovativeness was defined as nursing students’ voluntary willingness to attempt and enjoy the use of new medical technologies. To align with nursing students' characteristics, we adopted and modified two items from the innovativeness instrument (e.g., “I prefer using new technologies.”) [32]. The Cronbach’s alpha was 0.784. Digital literacy toward ICT for learning was assessed via an adapted digital literacy survey tool [27] tailored for this study, which comprised eight items (e.g., “I can learn new technologies easily.”). Cronbach’s alpha was 0.907. The responses of self-efficacy, innovativeness, and digital literacy were provided on a 5-point Likert scale ranging from “Strongly disagree” (1) to “Strongly agree” (5).

#### Technology acceptance factors

Perceived usefulness was measured via three items adapted from a tool used in a previous study [33], adjusted to suit nursing students' characteristics(e.g., “I expect that using telenursing in my job will increase my productivity.”). Cronbach’s alpha was 0.875. Perceived ease of use was assessed with two modified items (e.g., “I expect that telenursing systems will be easy to use.”). Cronbach's alpha was 0.881. Social influence comprised four items [34] tailored to nursing students (e.g., “People who influences my behaviors (seniors, juniors, and peers) will think that I should use telenursing system.”). Cronbach’s alpha was 0.812. Facilitation conditions, which reflected an individual's belief in organizational and technical support for utilizing new information systems, were measured using four items adapted to suit our target population [34] (e.g., “The telenursing system is not compatible with existing medical information systems.”) Cronbach’s alpha was 0.788. All items were rated on a 5-point Likert scale.

#### Perceptions of telenursing

Perception of telenursing was influenced by various factors, such as experience, specific conditions treated, and individual preferences [35]. In this study, perceptions of telenursing were defined as awareness of and experience with telenursing, educational exposure, and observation of telenursing during clinical practice. We included three items that examined telenursing experiences (e.g., “Do you have telenursing experience?”), five items that addressed telenursing education (e.g., “Have you received education on telenursing?”), and six items that evaluated perceptions and other individual understandings associated with telenursing (e.g., “Which way do you think is more satisfactory, telenursing or face-to-face nursing?”). In addition, one item assessed participants' awareness of telenursing by asking whether they know telenursing.

#### Attitude toward telenursing

Attitude toward telenursing was assessed via six items adapted from the tool utilized in previous research [33] and tailored to nursing students' characteristics(e.g., “Using telenursing is a good idea for nurses.”). The responses were provided on a 5-point Likert scale ranging from “Strongly disagree” (1) to “Strongly agree” (5). Cronbach’s alpha was 0.852.

### Data collection

The survey, conducted via Google Mobile Survey, adopted a self-reported approach and spanned from August 1 to September 31, lasting for two months. Convenience sampling was employed by recruiting participants through campus community notices and online postings.

### Data analysis

Collected data were analyzed via SPSS/WIN version 26.0. Participants’ demographic characteristics and their perceptions of telenursing were described as frequencies and percentages. The main variables were analyzed and described as means and standard deviations. Normal distribution was confirmed via the normality test. Differences in attitudes toward telenursing based on participants’ general characteristics and perceptions of telenursing were analyzed via a chi-squared test, independent t-test, and one-way analysis of variance (ANOVA). Pearson’s correlation coefficient was used to examine the correlation between the main variables. Multiple regression analysis was conducted to identify the factors that influenced attitudes toward telenursing. To assess the normality, homoscedasticity, and linearity of residuals, histograms and normal probability plots (P-P plots) of standardized residuals, as well as scatter plots, were examined.

### Ethical considerations

This research was approved by the Institutional Review Board of Seoul National University on July 2, 2023 (IRB No. 2307/001–012). The study's purpose was thoroughly explained via the mobile questionnaire to ensure participants' understanding of the consent form. Only individuals who fully comprehended the study and consented to participate were invited to respond to the survey. The authors highlighted the assurance of maintaining participants’ confidentiality and anonymity. A mobile gift card was presented to the participants who completed the online survey as a token of appreciation and to increase the reliability of the survey responses. All procedures were performed in accordance with the relevant guidelines and regulations.

## Results

### Participants’ general characteristics

This study included 188 participants. Table 1 presents the participants’ general characteristics. Among the participants, there were 174 females (92.6%) and 14 males (7.4%), which indicated a higher proportion of females. Participants were aged 18–47 years (average age: 22.49 ± 4.20 years).

**Table 1:** Participants’ general characteristics (*N* = 188)

Characteristics	Categories	N	%	M ± SD
Sex	Female	174	92.6	
	Male	14	7.4	
Age (Years)	17 < – ≤ 20	52	27.7	22.49 ± 4.20
	20 < – ≦24	106	56.4	
	24 < – ≦29	18	9.6	
	29 <	12	6.4	
Academic year	1st	21	11.2	
	2nd	56	29.8	
	3rd	56	29.8	
	4th	55	29.3	
Residential area	Metropolitan Area	95	50.5	
	Non-Metropolitan Area	93	49.5	
Received Clinical practice	Yes	120	63.8	
	No	68	36.2	

### Perceptions of telenursing

Table 2 presents the results regarding perceptions of telenursing. Regarding awareness of telenursing, 11.2%, 23.4%, 48.4%, and 17.0% claimed to be “very knowledgeable,” “knowledgeable,” “Heard of but not knowledgeable,” and “unknowledgeable,” respectively. Essentially, 65.4% lacked substantial awareness of telenursing and 19.1% had prior experience with telenursing. Of those who underwent clinical practice, 18.3% stated they observed telenursing practices at their clinical sites. We analyzed responses from participants with telenursing experience and found that 86.2% encountered the real-time or synchronous model. Most reported instances of consultations via telephone, particularly during the COVID-19 period. Cases of communication via telephone with isolated patients were reported in Residential Treatment Centers and negative pressure isolation rooms within hospital wards. In addition, two cases were reported for the “store & forward” or asynchronous type, which involved a later reporting of blood glucose and test results to nurses via home devices and transmission of health-related data through monitoring devices at home. For the “telemonitoring” or “remote monitoring” type, tracking exercise routes and times via GPS during health center exercise sessions (one case) and monitoring activity levels through sensor-equipped watches (one case) were reported. Finally, for the “mobile health” type, reports included responding to mobile health questionnaires via applications and daily health status reporting (three cases) and engagement in an artificial intelligence-internet of things–-based diabetes management program (one case). This study investigated the satisfactory way of delivering healthcare and found that 72.3% favored face-to-face nursing and 13.3% preferred telenursing, while 14.4% chose both. Similarly, regarding delivering healthcare, 75.5% opted for face-to-face, 14.9% chose telenursing, and 9.6% preferred both. Of the participants, 90.4% expressed an optimistic viewpoint regarding the prospect of telenursing. Among those who responded “pessimistic” toward the prospects of telenursing, specific opinions were solicited. Responses included various considerations, such as “considered nursing as requiring direct contact,” “difficult for older adults to use,” and “lack of accessibility for low-income and rural areas.”

**Table 2:** Perceptions of telenursing (*N* = 188)

Questions	Answers	N	%	Case Percent
Awareness of telenursing	Very knowledgeable	21	11.2	
	Knowledgeable	44	23.4	
	Heard of but not knowledgeable	91	48.4	
	Unknowledgeable	32	17.0	
Experience of telenursing	Yes	36	19.1	
	No	152	80.9	
Experienced telenursing type (Participants with telenursing experience only, *N* = 29)	Real time or synchronous(telephone)	25	75.6	86.2
	Store & forward or asynchronous	2	6.06	6.90
	Telemonitoring or remote monitoring	2	6.06	6.90
	Mobile health	4	12.1	13.8
Telenursing observation in Clinical practice (Participants with clinical practice, *N* = 120)	Yes	22	18.3	11.7
	No	98	81.7	52.1
Satisfactory way of delivering healthcare for patients	Face to Face	136	72.3	
	Telenursing	25	13.3	
	Both	27	14.4	
Preferred way of delivering healthcare for respondents	Face to Face	142	75.5	
	Telenursing	28	14.9	
	Both	18	9.6	
Nursing fields appropriate for telenursing application (Multiple responses)	Chronic Disease Care	101	18.1	53.7
	Home Health Care	109	19.6	58.0
	Hospice Care	32	5.7	17.0
	Discharge Care	145	26.0	77.1
	Palliative Care	46	8.2	24.5
	Health Education and Counseling	124	22.2	66.0
Preferred technological type of telenursing	Real time or synchronous	71	37.8	
	Store & forward or asynchronous	14	7.4	
	Telemonitoring or remote monitoring	67	35.6	
	Mobile health	36	19.1	
Preferred device for telenursing	Mobile phone	97	51.6	
	Telehealth specialized device	34	18.1	
	Wearable device	56	29.8	
	Others	1	0.5	
Prospects for telenursing (4.29 ± 0.682)	Very optimistic	76	40.4	
	Optimistic	94	50.0	
	Uncertain	15	8.0	
	Pessimistic	3	1.4	
	Very pessimistic	0	0	

### Status of education regarding telenursing

Table 3 presents the status of education regarding telenursing. This study investigated education exposure to telenursing and found that 18.6% had undergone telenursing education, while 81.4% had not. The most prevalent source of information for telenursing was the Internet, constituting 28.7%, followed by mass media and formal educational programs, both of which accounted for 18.5%. Furthermore, 14.7% reported learning telenursing through social media platforms, such as YouTube, Facebook, and Instagram, while 3.0% acquired knowledge from academic books or other literature. Additionally, 15.5% stated they were unaware of telenursing. In cases where respondents had experienced telenursing education, the types of education included regular academic programs (45.3%), one-time sessions, such as seminars or conferences (18.9%), and additional information provided by instructors outside the curriculum (35.9%). When the necessity and interest in telenursing education were assessed, necessity scored an average of 4.26 ± 0.702 out of 5, with a 0% response rate for “disagree” or “strongly disagree” options. Interest in telenursing education averaged 4.04 ± 0.786 out of 5, and no respondents selected “not at all” Differences in digital literacy based on the type of education experience of telenursing revealed statistically significant higher levels of digital literacy among those who received education through regular academic programs (F = 2.058, *p* = 0.041) (Additional file 1).

**Table 3:** Status of education regarding telenursing (*N* = 188)

Questions	Answer	N	%	Case Percent
Exposure of telenursing education	Yes	35	18.6	
	No	153	81.4	
Ways of learning about telenursing (Multiple responses)	Internet	76	28.7	40.4
	Social Media Platform (YouTube, Facebook, Instagram etc.)	39	14.7	20.7
	Mass Media (Newspaper, Radio, TV etc.)	49	18.5	26.1
	Formal education in nursing major	49	18.5	26.1
	Reading material related to nursing majors (textbook, research paper etc.)	8	3.0	4.3
	Others	3	1.1	1.6
	None	41	15.5	21.8
Type of education experience of telenursing (Multiple choices)	Regular academic program	24	45.3	12.8
	One-time sessions (Seminar, Conference, Webinar etc.)	10	18.9	5.3
	Additional information beyond the curriculum	19	35.9	10.1
	None	145	-	77.1
Necessity of education of telenursing (4.26 ± 0.702)	Strongly Agree	64	34.0	
	Agree	108	57.4	
	Neutral	16	8.5	
	Disagree	0	0	
	Strongly disagree	0	0	
Interest in education of telenursing (4.04 ± 0.786)	Very much so	55	29.3	
	Yes	93	49.5	
	Not sure	33	17.6	
	No	7	3.7	
	Not at all	0	0	

### Descriptive statistics of the main variables

Table 4 shows the main variables rated on a 5-point Likert scale. We compared the variables’ mean values (obtained by dividing the total score of each variable by the number of items) and found that attitude toward telenursing and perceived ease of use had the highest and lowest mean scores of 4.06 ± 0.61 and 3.57 ± 0.98, respectively. Attitude toward telenursing, measured with six items on a 5-point scale, had an average score of 24.35 ± 3.677.

**Table 4:** Descriptive statistics of the main variables (*N* = 188)

Variables	Min	Max	Total Mean ± SD	Item Mean ± SD
Attitude toward telenursing	10	30	24.35 ± 3.677	4.06 ± 0.61
Perceived usefulness	3	15	11.68 ± 2.375	3.89 ± 0.79
Perceived ease of use	2	10	7.14 ± 1.968	3.57 ± 0.98
Social influences	8	20	15.11 ± 2.750	3.78 ± 0.69
Facilitating conditions	7	20	14.86 ± 2.789	3.71 ± 0.70
Self-efficacy	4	10	8.02 ± 1.555	4.01 ± 0.78
Innovativeness	2	10	7.52 ± 1.857	3.76 ± 0.93
Digital literacy	11	40	30.68 ± 5.439	3.83 ± 0.70

### Attitude toward telenursing and main variables

An independent t-test and one-way ANOVA were performed to investigate the factors that influenced attitudes toward telenursing and main variables according to participants’ general characteristics (Additional file 2) and perceptions of telenursing (Table 5). Attitudes toward telenursing significantly differed regarding experiences of telenursing (t = 2.746, *p* = 0.007) and telenursing observations in clinical practice (t = 4.002, *p* < 0.000). No significant differences were observed among sex (t = -1.070, *p* = 0.286), age groups (F = 2.589, *p* = 0.054), academic year (F = 1.449, *p* = 0.230), residential area (t = 1.479, *p* = 0.141), or clinical practice experiences (t = 0.502, *p* = 0.616). Post-hoc Scheffe’s test concerning attitudes toward telenursing with age groups revealed significantly higher scores for the “24 < N≦29” group compared with the “29 < N” group (*p* = 0.023). Furthermore, there was a tendency for telenursing awareness to influence attitudes toward nursing (F = 2.482, *p* = 0.062); however, it was not statistically significant. Participants with telenursing experience and those with telenursing observations in clinical practice showed significantly higher scores in most variables, which included attitudes toward nursing. The group exposed to education in telenursing showed significant differences in all the variables.

**Table 5:** Differences in main variables according to perceptions of telenursing (*N* = 188)

Variables	Categories	N	PU		PEU		SI		FC		SE		IV		DL		ATT	
			M ± SD	F of t(p)	M ± SD	F of t(p)	M ± SD	F of t(p)	M ± SD	F of t(p)	M ± SD	F of t(p)	M ± SD	F of t(p)	M ± SD	F of t(p)	M ± SD	F of t(p)
Experience of telenursing	Yes	36	4.10 ± 0.73	1.765 (.079)	3.83 ± 1.01	1.802 (.073)	4.06 ± 0.83	2.320 (.025)*	3.95 ± 0.78	2.284 (.024)*	4.28 ± 0.58	2.320 (.021)*	4.08 ± 0.74	2.760 (.007)*	4.23 ± 0.53	4.029 (< .001)***	4.31 ± 0.60	2.746 (.007)**
	No	152	3.84 ± 0.80		3.51 ± 0.97		3.71 ± 0.63		3.66 ± 0.67		3.95 ± 0.81		3.68 ± 0.95		3.74 ± 0.68		4.00 ± 0.60	
Telenursing observation in Clinical Practice	Yes	22	4.33 ± 0.47	4.072 (< .001)***	4.00 ± 1.04	1.870 (.064)	4.31 ± 0.61	4.375 (< .001)***	4.13 ± 0.73	3.136 (.002)**	4.20 ± 0.70	1.650 (.102)	4.14 ± 0.66	2.546 (.015)*	4.28 ± 0.55	3.823 (< .001)***	4.51 ± 0.42	4.002 (< .001)***
	No	98	3.80 ± 0.83		3.58 ± 0.94		3.65 ± 0.64		3.61 ± 0.69		3.92 ± 0.73		3.70 ± 0.95		3.70 ± 0.68		3.98 ± 0.58	
Telenursing Education exposure	Yes	35	4.27 ± 0.52	4.182 (< .001)***	4.00 ± 0.91	2.929 (.004)*	4.12 ± 0.63	3.365 (.001)*	4.01 ± 0.70	2.865 (.005)*	4.27 ± 0.57	2.223 (.027)*	4.19 ± 0.76	.098 (.002)**	4.11 ± 0.58	.635 (.007)**	4.32 ± 0.60	2.904 (.004)**
	No	153	3.81 ± 0.82		3.47 ± 0.98		3.70 ± 0.68		3.65 ± 0.68		3.95 ± 0.81		3.66 ± 0.94		3.77 ± 0.69		4.00 ± 0.60	

^*^PU Perceived Usefulness, *PEU* Perceived Ease of Use, *SI* Social Influences, *FC* Facilitating Conditions, *SE* Self-Efficacy, *IV* Innovativeness, *DL* Digital Literacy, *ATT* Attitude Toward Telenursing, *p<.05, **p<.01, ***p<.001

### Correlations between the main variables

Pearson’s correlation analysis was conducted to examine the correlations between the main variables (Table 6). A statistically significant positive correlation was confirmed for each main variable. Specifically, social influences exhibited strong correlations with perceived usefulness (*r* = 0.697, *p* < .001), facilitating conditions (*r* = 0.689, *p* < .001), and attitudes toward telenursing (*r* = 0.693, *p* < .001). Additionally, perceived usefulness showed a significant correlation with attitudes toward telenursing (*r* = 0.675, *p* < .001). Innovativeness was significantly correlated with digital literacy (*r* = 0.693, *p* < .001), and correlation coefficients ranged from 0.65–0.7.

**Table 6:** Correlations between the main variables (*N* = 188)

Variables	1	2	3	4	5	6	7	8
1. Perceived usefulness	1							
2. Perceived ease of use	.541***	1						
3. Social influences	.697***	.628***	1					
4. Facilitating conditions	.501***	.553***	.689***	1				
5. Self-efficacy	.402***	.401***	.540***	.552***	1			
6. Innovativeness	.344***	.331***	.394***	.415***	.606***	1		
7. Digital literacy	.311***	.292***	.430***	.473***	.583***	.693***	1	
8. Attitude toward telenursing	.675***	.523***	.693***	.530***	.519***	.455***	.420***	1

^*^p < .05

^**^p < .01

^***^p < .001

### Factors that affected the attitude toward telenursing

A multiple regression analysis was performed to identify the factors that affected the attitude toward telenursing. Among the individual factors that led to significant differences in participants’ attitudes toward telenursing, “experience of telenursing” and “exposure to telenursing education” were selected as independent variables. Although the telenursing observation in clinical practice significantly influenced attitudes toward telenursing, further cross-analysis via a chi-squared test demonstrated a strong correlation with experiences of telenursing (*p* < .001). Consequently, this variable was subsequently excluded from the analysis.

**Fig. 2:**
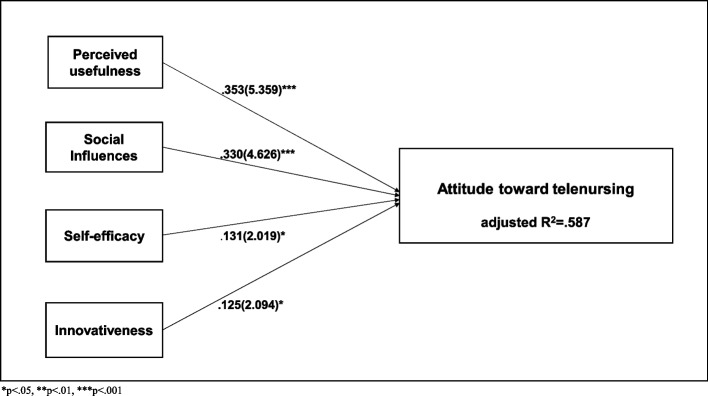
Associated factors of attitude toward telenursing

Table 7 presents the results of the multiple regression analysis. The regression model demonstrated statistical significance (F = 67.445, *p* < .001). The adjusted R2, which represented the model’s explanatory power, was 0.587. The Durbin-Watson statistic of 2.131 was approximately 2, which indicated that there was no problem with assuming the independence of the residuals. Additionally, all variance inflation factors were below 10, which indicated no multicollinearity problems. Significant coefficients were found for perceived usefulness (β = 0.353), social influence (β = 0.330), self-efficacy (β = 0.131), and innovativeness (β = 0.125), which revealed a positive influence on attitudes toward telenursing. Figure 2 depicts the factors that affected the attitude toward telenursing.

**Table 7:** Associated factors of attitude toward telenursing (*N* = 188)

Variables	*β*	t	p	TOL	VIF
Exposure to telenursing education (Yes)	-.011	-.221	.825	.918	1.090
Experience of telenursing (Yes)	.037	.776	.439	.950	1.053
Perceived usefulness	.353	5.359	< .000	.508	1.968
Perceived ease of use	.043	.696	.487	.577	1.732
Social influences	.330	4.626	< .000	.433	2.308
Facilitating conditions	.006	.089	.929	.476	2.099
Self-efficacy	.131	2.019	.045	.526	1.968
Innovativeness	.125	2.094	.038	.621	1.611
Digital literacy	-.014	-.198	.843	.466	2.144

F = 67.445(*p* < .000), R^2^ = .596, adjusted R^2^ = .587, Durbin-Watson = 2.131

Referent group of dummy variables: Telenursing experience “No,” Telenursing education experience “No”

## Discussion

Our findings revealed that nursing students demonstrated a lack of substantial awareness of telenursing; however, they also simultaneously displayed a positive outlook. Although the current provision of telenursing education was severely deficient, majority of the students recognized its necessity and showed high interest in telenursing education. Nursing students' attitudes toward telenursing were independent of demographic factors. However, it was associated with telenursing experience and education and observation of telenursing during clinical practice. These factors showed notable associations with attitudes and most of the main variables, such as perceived usefulness, perceived ease of use, social influences, facilitating conditions, innovativeness, self-efficacy, and digital literacy. In particular, the four factors, perceived usefulness, social influences, self-efficacy, and innovativeness, significantly influenced attitudes toward telenursing sequentially.

### Perceptions of telenursing

Most nursing students demonstrated a lack of substantial awareness regarding telenursing. Despite their notably positive outlook on telenursing, the preferred and prioritized method of healthcare delivery was face-to-face nursing. This indicated that they had limited receptiveness toward it. Previous studies on nursing students also reported simultaneous positive prospects alongside negative perceptions toward telenursing, which were associated with a lack of awareness [20, 36]. Prior research findings also indicated that the negative perceptions stemmed from concerns, such as reduced patient-nurse interaction, impracticality, service inconvenience, and apprehensions regarding service imbalances [36, 37]. In this study, a similar finding was observed as participants who held negative prospects of telenursing also mentioned its impracticality compared to face-to-face nursing, lack of patient contact, challenges faced by older individuals, and accessibility issues for low-income or rural residents. However, such negative opinions were a contrast to those of recent research that indicated telenursing resolved various issues of face-to-face nursing, surpassed spatial and temporal constraints to enhance accessibility, and demonstrated high satisfaction levels [38]. This limited perception was presumed to stem from the absence of formal education in telenursing [39]. Another previous study reported the availability of technology, access to the Internet, and lack of telemedicine training as the most significant factors that influenced healthcare providers' perception of telenursing [40]. Hence, providing nursing students with telenursing education could serve as a significant facilitator in understanding and harnessing it [20]. However, only one-fifth of the respondents reported education in telenursing, even though the students recognized its necessity and exhibited a strong interest.

### Factors affecting attitude toward telenursing

Attitudes of nursing students toward telenursing showed a significant association with telenursing experience, observation of telenursing during clinical practice, and exposure to telenursing education. These experiences notably influenced their perception and willingness toward telenursing [17]. Additionally, these factors demonstrated associations with crucial variables, such as perceived usefulness, ease of use, social influences, facilitating conditions, innovativeness, self-efficacy, and digital literacy. These variables affected attitudes and also influenced intentions to use and actual future utilization. Lack of knowledge and awareness could impede the recognition and utilization of telehealth services and potentially hinder the broader adoption of these advancements [41]. These insights underscore the need to implement programs aimed at augmenting knowledge and practical exposure. Through such initiatives, we can markedly enhance their understanding and readiness to embrace telenursing.

In particular, perceived usefulness, social influences, innovativeness, and self-efficacy were identified as key determinants in shaping nursing students' attitudes toward telenursing. Similar results were also identified in previous studies [33, 42]. Perceived usefulness, which referred to the belief in telenursing's capacity to enhance work performance [28], was the most significant factor that influenced attitudes toward telenursing. Telenursing offered various benefits, such as improved medical accessibility and efficient healthcare management, and recognizing these advantages exerted significant influence [38]. Social influences emerged as the second most influential factor that affected attitudes toward telenursing. Shared understanding among nursing students and senior nurses regarding the usefulness and necessity of utilizing telenursing could positively influence attitudes toward telenursing and also its intention and actual utilization [43]. This study's findings also emphasized the significance of individual factors, such as innovativeness and self-efficacy. These factors represented the importance of nursing students' perception of their capability and willingness to use telenursing. Previous studies suggested that voluntariness exerted the most significant influence on the intention to utilize telehealth services [12]. This implied that individual recognition and willingness held more significance in fostering the utilization of telehealth or telenursing than external environmental factors that could induce non-voluntary use. By equipping students with practical proficiency, they could develop personal aspects, such as innovativeness and self-efficacy.

### Implication and necessity of telenursing education

All the aforementioned findings emphasized the need for telenursing education. Nursing students need telenursing education as part of their preparation for future roles. Such education could enhance their knowledge and also cultivate a favorable attitude toward it [44]. Individuals exposed to telehealth education demonstrate a significantly improved understanding of its utility and role [45]. A nurse's expertise, which requires the seamless integration of various skills, such as information technology, nursing proficiency, and communication skills, should be cultivated through education [46, 47]. In addition, experiential education programs could augment crucial factors, such as perceived usefulness, self-efficacy, and innovativeness, by deepening understanding of telenursing's effectiveness and implications [47]. However, previous studies have reported the inadequacy of telehealth education within nursing programs [43]. Furthermore, the undergraduate nursing informatics curriculum was reported to be insufficient, and there was scarce information on telenursing education in Korean nursing universities [48]. The lack of nursing informatics education may result in challenges for nursing students in fostering information technology competencies, potentially leading to difficulties in comprehending and utilizing telenursing. Therefore, it is necessary to include practical telenursing education in the nursing curriculum. This ensures that nursing students have access to fundamental telenursing education to prepare them adequately for their forthcoming roles.. Additionally, the quantity and quality of nursing informatics education should be improved. Nursing informatics education would elevate their telenursing competency by equipping nursing students with the necessary knowledge and skills in information technology.

Further research should focus on the integration of formal education curricula on telenursing and the provision of experiential-based learning opportunities, such as simulation education. Moreover, investigation into the intentions and actual usage of telenursing among nursing students transitioning into healthcare professionals is warranted. Such research endeavors can contribute to establishing effective education on telenursing, initiating educational efforts promptly, and advocating to raise awareness of its benefits.

### Strengths and limitations

This study had several limitations. First, the participants could have had pre-existing positive or negative opinions of telenursing, which could have influenced their responses. Second, we focused only on factors that influenced attitudes toward telenursing and did not directly explain whether these had an impact on the intention or actual usage of telenursing. Third, the participants were selected through convenience sampling. Hence, our results should be interpreted and generalized with caution.

However, despite these limitations, this study was significant as the first investigation into the perceptions and attitudes of Korean nursing students toward telenursing following the COVID-19 pandemic. Previous studies predominantly targeted healthcare professionals and telehealth, which left a significant gap in research focusing on telenursing among nursing students. This study highlighted the status of nursing students concerning telenursing and provided insights into the necessary preparations for nurturing future telenursing professionals.

## Conclusion

This study revealed that nursing students had limited awareness of telenursing. However, they displayed a positive outlook toward it. The students' attitudes toward telenursing were associated not only with factors such as perceived usefulness, social influence, self-efficacy, and innovativeness but also with their experiences in telenursing, observations during clinical practice, and exposure to telenursing education. Ultimately, telenursing education played a crucial role in the development of specialized knowledge required for clinical telenursing practice. Nursing students require access to formal telenursing education to prepare for their future nursing roles. This education enhances students' competency and also nurtures a positive attitude conducive to the seamless integration of telenursing into the forthcoming digital healthcare era.

### Supplementary Information

**Additional file 1: Table S1.** Differences in digital literacy based on the type of education experience of telenursing.

**Additional file 2: Table S2.** Differences in the main variables according to general characteristics.

## Data Availability

Datasets used and analyzed in this study are available from the corresponding author upon reasonable request.

## Abbreviations

ANOVA: Analysis of Variance
TAM: Technology Acceptance Model
