# Supplementary material for: Investigating perceptions and attitude toward telenursing among undergraduate nursing students for the future of nursing education: a cross-sectional study
Source: BMC Nurs. 2024 Apr 8;23:236. doi: 10.1186/s12912-024-01903-2 (PMC11000379; doi:10.1186/s12912-024-01903-2)
Supplement: Supplementary file 1 — Additional file 1: Table S1. Differences in digital literacy based on the type of education experience of telenursing. [file 12912_2024_1903_MOESM1_ESM.pdf]

Table S1. Differences in digital literacy based on the type of education experience of telenursing

Type of education experience about telenursing		Regular academic program		One-time sessions (seminar, conference, webinar)		Additional information beyond the curriculum	
		Yes	No	Yes	No	Yes	No
Variables	N	24	164	10	178	19	169
Digital literacy	Mean±SD	4.10±0.65	3.80±0.68	4.14±0.55	3.82±0.68	3.73±0.51	3.85±0.70
	F of t(p)	2.058(.041)		1.453(.148)		-.704(.483)	
