# Supplementary material for: Investigating perceptions and attitude toward telenursing among undergraduate nursing students for the future of nursing education: a cross-sectional study
Source: BMC Nurs. 2024 Apr 8;23:236. doi: 10.1186/s12912-024-01903-2 (PMC11000379; doi:10.1186/s12912-024-01903-2)
Supplement: Supplementary file 2 — Additional file 2: Table S2. Differences in the main variables according to general characteristics. [file 12912_2024_1903_MOESM2_ESM.pdf]

Table S2. Differences in the main variables according to general characteristics

(N =188)

Variables	Categories	N	PU		PEU		SI		FC		SE		IV		DL		ATT	
			M±SD	F of t(p)	M±SD	F of t(p)	M±SD	F of t(p)	M±SD	F of t(p)	M±SD	F of t(p)	M±SD	F of t(p)	M±SD	F of t(p)	M±SD	F of t(p)
Sex	Female	174	3.89±0.78	-.522(.603)	3.55±0.98	-.714(.476)	3.78±0.68	-.044(.965)	3.72±0.68	.006(.995)	3.99±0.78	-1.561(.120)	3.72±0.93	-1.772(.078)	3.81±0.67	-1.618(.107)	4.04±0.61	-1.070(.286)
	Male	14	4.00±0.92		3.75±1.03		3.79±0.81		3.71±0.92		4.32±0.72		4.18±0.89		4.12±0.74		4.23±0.62	
Age	17< – ≤20	52	3.94±0.71	1.199(.312)	3.44±1.02	0.709(.548)	3.78±0.61	0.316(.814)	3.73±0.60	0.405(.750)	4.02±0.82	0.660(.578)	3.72±0.92	0.087(.967)	3.82±0.63	0.030(.993)	4.06±0.58	2.589(.054)
	20< – ≦24	106	3.84±0.85		3.63±0.97		3.78±0.70		3.73±0.74		3.96±0.77		3.77±0.94		3.84±0.68		4.06±0.64	
	24< – ≦29	18	4.19±0.59		3.72±0.81		3.85±0.67		3.72±0.67		4.19±0.79		3.83±0.95		3.86±0.86		4.28±0.52	
	29<	12	3.75±0.87		3.38±1.19		3.60±0.92		3.50±0.83		4.17±0.72		3.75±0.89		3.79±0.72		3.65±0.55	
Academic year	1st[a]	21	3.73±0.66		3.29±0.72		3.67±0.55		3.64±0.47		3.81±0.68		3.71±0.75		3.86±0.58		3.90±.0.51	
	2nd[b]	56	4.05±0.81		3.58±1.16		3.96±0.80		3.90±0.76		4.21±0.88		3.83±1.00		4.00±0.68		4.14±0.71	1.449(.230)

	3rd[c]	56	3.88±0.87	1.208(.308)	3.61±0.99	0.671(.571)	3.76±0.66	2.021(.113)	3.74±0.67	2.775(.043)*	3.93±0.74	2.060(.107)	3.71±0.99	.171(.916)	3.76±0.70	1.672(.175)	4.12±0.62	
	4th[d]	55	3.81±0.72		3.63±0.88		3.66±0.62		3.53±0.70	(b>d)	3.96±0.71		3.75±0.87		3.74±0.68		3.97±0.52	
Residential area	Metropolitan area	95	3.94±0.76	.879(.381)	3.59±1.03	.359(.720)	3.87±0.69	1.781(.077)	3.77±0.69	1.001(.318)	4.06±0.80	.936(.350)	3.86±0.93	.1.576(.117)	3.96±0.71	2.582(.011)*	4.12±0.63	1.479(.141)
	Non-Metropolitan area	93	3.84±0.82		3.54±0.94		3.69±0.67		3.66±0.70		3.96±0.75		3.65±0.92		3.71±0.63		3.99±0.60	
Clinical Practice	Yes	120	3.90±0.81	.083(.934)	3.65±0.97	1.580(.116)	3.78±0.68	-.187(.852)	3.70±0.72	-.293(.770)	3.98±0.73	-.834(.405)	3.78±0.91	.496(.620)	3.80±0.69	-.922(.358)	4.08±0.59	0.502(.616)
	No	68	3.89±0.77		3.42±1.00		3.79±0.70		3.74±0.66		4.07±0.86		3.71±0.96		3.90±0.65		4.02±0.66	

Note: *PU=Perceived Usefulness, PEU=Perceived Ease of Use, SI=Social Influences, FC=Facilitating Conditions, SE=Self-Efficacy, IV=Innovativeness, DL=Digital Literacy, ATT=Attitude Toward Telenursing
